# 3,3′-Dinitro-4,4′-bipyridine

**DOI:** 10.1107/S1600536811014139

**Published:** 2011-04-22

**Authors:** Yong Wang, Jing-Yi Xu, De-Yong Li, Lu Shi

**Affiliations:** a Department of Chemical Engineering, Henan Polytechnic Institute, Nanyang 473009, People’s Republic of China; b Pingdingshan Research Institute of Functional Materials, Pingdingshan 467000, People’s Republic of China; c Department of Applied Chemistry, College of Science, Nanjing University of Technology, Nanjing 210009, People’s Republic of China

## Abstract

In the title compound, C_10_H_6_N_4_O_4_, the pyridine rings are oriented at a dihedral angle of 67.8 (1)°. The O-atom pairs are *trans*, each displaced by a similar distance [average = 0.2331 (2) Å] out of the attached pyridine ring plane. In the crystal, inter­molecular C—H⋯O and C—H⋯N inter­actions link the mol­ecules into a three-dimensional network.

## Related literature

For applications of the title compound, see: Katritzky *et al.* (2006 ▶). For the synthesis, see: Kaczmarek *et al.* (1980 ▶). For bond-length data, see: Allen *et al.* (1987 ▶).
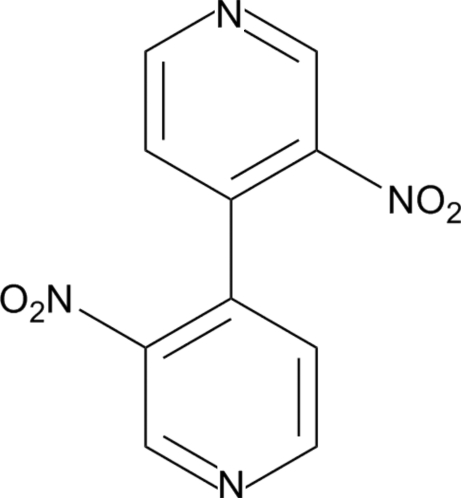


## Experimental

### 

#### Crystal data

C_10_H_6_N_4_O_4_
*M* *_r_* = 246.19
Orthorhombic, 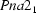

*a* = 9.3580 (19) Å
*b* = 17.815 (4) Å
*c* = 6.3870 (13) Å
*V* = 1064.8 (4) Å^3^
*Z* = 4
Mo *K*α radiation
μ = 0.12 mm^−1^
*T* = 293 K
0.20 × 0.10 × 0.10 mm

#### Data collection

Enraf–Nonius CAD-4 diffractometer
Absorption correction: ψ scan (North *et al.*, 1968 ▶) *T*_min_ = 0.976, *T*_max_ = 0.988
2089 measured reflections
1071 independent reflections
679 reflections with *I* > 2σ(*I*)
*R*_int_ = 0.042
3 standard reflections every 200 reflections  intensity decay: 1%

#### Refinement

*R*[*F* ^2^ > 2σ(*F* ^2^)] = 0.055
*wR*(*F* ^2^) = 0.141
*S* = 1.00
1071 reflections
163 parameters
1 restraint
H-atom parameters constrained
Δρ_max_ = 0.18 e Å^−3^
Δρ_min_ = −0.22 e Å^−3^

### 

Data collection: *CAD-4 Software* (Enraf–Nonius, 1985 ▶); cell refinement: *CAD-4 Software*; data reduction: *XCAD4* (Harms & Wocadlo, 1995 ▶); program(s) used to solve structure: *SHELXS97* (Sheldrick, 2008 ▶); program(s) used to refine structure: *SHELXL97* (Sheldrick, 2008 ▶); molecular graphics: *SHELXTL* (Sheldrick, 2008 ▶); software used to prepare material for publication: *SHELXTL*.

## Supplementary Material

Crystal structure: contains datablocks I, global. DOI: 10.1107/S1600536811014139/bq2296sup1.cif

Structure factors: contains datablocks I. DOI: 10.1107/S1600536811014139/bq2296Isup2.hkl

Supplementary material file. DOI: 10.1107/S1600536811014139/bq2296Isup3.cml

Additional supplementary materials:  crystallographic information; 3D view; checkCIF report

## Figures and Tables

**Table 1:** Hydrogen-bond geometry (Å, °)

*D*—H⋯*A*	*D*—H	H⋯*A*	*D*⋯*A*	*D*—H⋯*A*
C2—H2*B*⋯O1^i^	0.93	2.40	3.234 (8)	149
C3—H3*A*⋯N2^ii^	0.93	2.62	3.440 (8)	147
C10—H10*A*⋯O2^iii^	0.93	2.57	3.392 (6)	148

Symmetry codes: (i) 
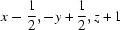
; (ii) 
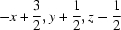
; (iii) 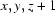.

**Fig. 1.**
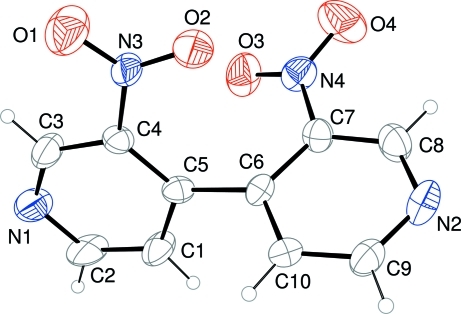
The molecular structure of (I), with the atom-numbering scheme. Displacement ellipsoids are drawn at the 50% probability level.

**Fig. 2.**
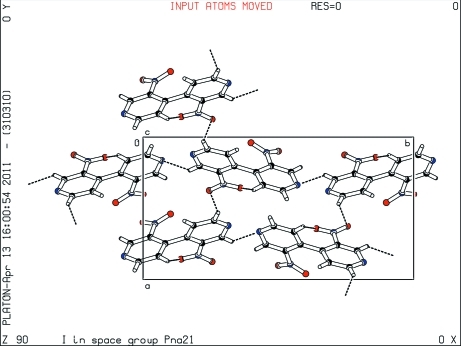
A packing diagram of (I), viewed down c-axis. Hydrogen bonds are shown as dashed lines.

## supplementary crystallographic information

### Comment

The tittle compound, 3,3'-dinitro-4,4'-bipyridine is an important intermediate (Katritzky *et al.*, 2006) and we report here the crystal structure of the title compound, (I).

The molecular structure of (I) is shown in Fig. 1, and the intermolecular C—H···O and C—H···N hydrogen bonds (Table 1) result in the molecular packing in three dimension (Fig. 2.). The bond lengths and angles are within normal ranges (Allen *et al.*, 1987).

In the molecule of the title compound, the dihedral angle of the pyridine rings [(C1-C5/N1) and (C6-C10/N2)] is 67.8 (1)°.

In the crystal structure, intermolecular C—H···O and C—H···N interactions link the molecules.

### Experimental

The title compound, (I) was prepared by the method of Ullmann reaction reported in literature (Kaczmarek *et al.* (1980). The crystals were obtained by dissolving (I) (0.2 g, 0.81 mmol) in ethanol (25 ml) and evaporating the solvent slowly at room temperature for about 5 d.

### Refinement

H atoms were positioned geometrically and refined as riding groups, with C—H = 0.93 Å for aromatic H, and constrained to ride on their parent atoms, with *U*_iso_(H) = *1.2U*_eq_(C).

### Crystal data

**Table:**

C_10_H_6_N_4_O_4_	*F*(000) = 504
*M**_r_* = 246.19	*D*_x_ = 1.536 Mg m^−^^3^
Orthorhombic, *P**n**a*2_1_	Mo *K*α radiation, λ = 0.71073 Å
Hall symbol: P 2c -2n	Cell parameters from 25 reflections
*a* = 9.3580 (19) Å	θ = 9–13°
*b* = 17.815 (4) Å	µ = 0.12 mm^−^^1^
*c* = 6.3870 (13) Å	*T* = 293 K
*V* = 1064.8 (4) Å^3^	Block, yellow
*Z* = 4	0.20 × 0.10 × 0.10 mm

### Data collection

**Table:**

Enraf–Nonius CAD-4 diffractometer	679 reflections with *I* > 2σ(*I*)
Radiation source: fine-focus sealed tube	*R*_int_ = 0.042
graphite	θ_max_ = 25.4°, θ_min_ = 2.3°
ω/2θ scans	*h* = −11→0
Absorption correction: ψ scan (North *et al.*, 1968)	*k* = −21→21
*T*_min_ = 0.976, *T*_max_ = 0.988	*l* = −7→0
2089 measured reflections	3 standard reflections every 200 reflections
1071 independent reflections	intensity decay: 1%

### Refinement

**Table:**

Refinement on *F*^2^	Primary atom site location: structure-invariant direct methods
Least-squares matrix: full	Secondary atom site location: difference Fourier map
*R*[*F*^2^ > 2σ(*F*^2^)] = 0.055	Hydrogen site location: inferred from neighbouring sites
*wR*(*F*^2^) = 0.141	H-atom parameters constrained
*S* = 1.00	*w* = 1/[σ^2^(*F*_o_^2^) + (0.075*P*)^2^] where *P* = (*F*_o_^2^ + 2*F*_c_^2^)/3
1071 reflections	(Δ/σ)_max_ < 0.001
163 parameters	Δρ_max_ = 0.18 e Å^−^^3^
1 restraint	Δρ_min_ = −0.22 e Å^−^^3^

### Special details

**Table:**

Geometry. All e.s.d.'s (except the e.s.d. in the dihedral angle between two l.s. planes) are estimated using the full covariance matrix. The cell e.s.d.'s are taken into account individually in the estimation of e.s.d.'s in distances, angles and torsion angles; correlations between e.s.d.'s in cell parameters are only used when they are defined by crystal symmetry. An approximate (isotropic) treatment of cell e.s.d.'s is used for estimating e.s.d.'s involving l.s. planes.
Refinement. Refinement of *F*^2^ against ALL reflections. The weighted *R*-factor *wR* and goodness of fit *S* are based on *F*^2^, conventional *R*-factors *R* are based on *F*, with *F* set to zero for negative *F*^2^. The threshold expression of *F*^2^ > σ(*F*^2^) is used only for calculating *R*-factors(gt) *etc*. and is not relevant to the choice of reflections for refinement. *R*-factors based on *F*^2^ are statistically about twice as large as those based on *F*, and *R*- factors based on ALL data will be even larger.

### Fractional atomic coordinates and isotropic or equivalent isotropic displacement parameters (Å^2^)

**Table:**

	*x*	*y*	*z*	*U*_iso_*/*U*_eq_	
N1	0.6207 (7)	0.3139 (2)	0.2435 (8)	0.0921 (18)	
C1	0.6194 (6)	0.1867 (3)	0.3674 (10)	0.0729 (17)	
H1B	0.5890	0.1532	0.4699	0.088*	
O1	0.8746 (5)	0.2549 (2)	−0.2244 (9)	0.1052 (17)	
N2	0.8363 (5)	−0.0689 (2)	0.2211 (10)	0.0747 (15)	
O2	0.8568 (5)	0.1406 (2)	−0.1756 (6)	0.0897 (15)	
C2	0.5853 (7)	0.2620 (3)	0.3771 (10)	0.093 (2)	
H2B	0.5311	0.2772	0.4916	0.111*	
N3	0.8294 (4)	0.2037 (2)	−0.1255 (7)	0.0600 (12)	
O3	0.5400 (5)	0.1011 (2)	−0.1225 (8)	0.0950 (16)	
C3	0.7017 (6)	0.2911 (3)	0.0879 (10)	0.0721 (18)	
H3A	0.7333	0.3269	−0.0075	0.087*	
N4	0.6084 (5)	0.0441 (3)	−0.1202 (8)	0.0665 (12)	
O4	0.5987 (6)	0.0001 (3)	−0.2657 (8)	0.1145 (18)	
C4	0.7423 (5)	0.2184 (2)	0.0572 (8)	0.0491 (12)	
C5	0.7021 (5)	0.1625 (2)	0.1959 (8)	0.0461 (11)	
C6	0.7473 (5)	0.0821 (2)	0.1935 (8)	0.0504 (13)	
C7	0.7029 (5)	0.0261 (3)	0.0504 (9)	0.0518 (13)	
C8	0.7463 (6)	−0.0465 (3)	0.0707 (11)	0.0690 (16)	
H8A	0.7121	−0.0819	−0.0238	0.083*	
C9	0.8758 (6)	−0.0164 (3)	0.3553 (9)	0.0685 (15)	
H9A	0.9354	−0.0310	0.4645	0.082*	
C10	0.8368 (5)	0.0574 (3)	0.3476 (8)	0.0545 (13)	
H10A	0.8712	0.0908	0.4475	0.065*	

### Atomic displacement parameters (Å^2^)

**Table:**

	*U*^11^	*U*^22^	*U*^33^	*U*^12^	*U*^13^	*U*^23^
N1	0.150 (5)	0.066 (3)	0.060 (3)	0.037 (3)	0.023 (4)	0.003 (3)
C1	0.097 (4)	0.061 (3)	0.061 (4)	0.012 (3)	0.036 (4)	0.018 (3)
O1	0.108 (4)	0.090 (3)	0.118 (4)	−0.002 (3)	0.039 (3)	0.016 (3)
N2	0.077 (3)	0.052 (3)	0.094 (4)	0.004 (2)	−0.006 (3)	0.025 (3)
O2	0.123 (4)	0.074 (3)	0.073 (3)	0.015 (2)	0.055 (3)	−0.002 (2)
C2	0.141 (6)	0.090 (4)	0.046 (3)	0.039 (4)	0.054 (4)	0.007 (4)
N3	0.077 (3)	0.047 (2)	0.056 (3)	−0.005 (2)	0.030 (3)	0.003 (2)
O3	0.104 (3)	0.071 (2)	0.109 (4)	0.009 (2)	−0.049 (4)	0.010 (3)
C3	0.101 (5)	0.048 (3)	0.067 (4)	0.014 (3)	0.023 (4)	0.015 (3)
N4	0.062 (3)	0.071 (3)	0.066 (3)	−0.009 (2)	−0.013 (3)	0.009 (3)
O4	0.115 (4)	0.133 (4)	0.095 (3)	0.016 (3)	−0.050 (3)	−0.038 (4)
C4	0.057 (3)	0.049 (2)	0.041 (3)	0.011 (2)	0.006 (2)	−0.004 (2)
C5	0.043 (2)	0.056 (3)	0.039 (3)	0.005 (2)	0.007 (3)	0.001 (2)
C6	0.058 (3)	0.043 (2)	0.050 (3)	−0.006 (2)	0.004 (3)	0.011 (2)
C7	0.047 (3)	0.049 (3)	0.060 (3)	−0.004 (2)	−0.003 (3)	0.000 (3)
C8	0.078 (4)	0.054 (3)	0.074 (4)	−0.006 (3)	−0.012 (4)	−0.011 (3)
C9	0.085 (4)	0.056 (3)	0.065 (4)	0.005 (3)	−0.016 (4)	0.015 (3)
C10	0.067 (3)	0.056 (3)	0.040 (3)	−0.001 (2)	−0.002 (3)	0.006 (2)

### Geometric parameters (Å, °)

**Table:**

N1—C2	1.302 (7)	C3—H3A	0.9300
N1—C3	1.314 (7)	N4—O4	1.219 (6)
C1—C2	1.380 (7)	N4—C7	1.440 (7)
C1—C5	1.409 (8)	C4—C5	1.385 (6)
C1—H1B	0.9300	C5—C6	1.494 (6)
O1—N3	1.188 (5)	C6—C10	1.366 (7)
N2—C9	1.321 (7)	C6—C7	1.415 (6)
N2—C8	1.338 (8)	C7—C8	1.362 (6)
O2—N3	1.198 (5)	C8—H8A	0.9300
C2—H2B	0.9300	C9—C10	1.365 (7)
N3—C4	1.447 (6)	C9—H9A	0.9300
O3—N4	1.201 (5)	C10—H10A	0.9300
C3—C4	1.364 (6)		
			
C2—N1—C3	115.0 (4)	C5—C4—N3	122.6 (4)
C2—C1—C5	117.3 (5)	C4—C5—C1	115.3 (4)
C2—C1—H1B	121.3	C4—C5—C6	127.3 (4)
C5—C1—H1B	121.3	C1—C5—C6	117.2 (4)
C9—N2—C8	115.5 (4)	C10—C6—C7	114.7 (4)
N1—C2—C1	127.0 (5)	C10—C6—C5	118.4 (5)
N1—C2—H2B	116.5	C7—C6—C5	126.8 (5)
C1—C2—H2B	116.5	C8—C7—C6	121.4 (5)
O1—N3—O2	120.2 (5)	C8—C7—N4	117.8 (5)
O1—N3—C4	119.4 (4)	C6—C7—N4	120.8 (4)
O2—N3—C4	120.4 (4)	N2—C8—C7	122.6 (5)
N1—C3—C4	124.3 (5)	N2—C8—H8A	118.7
N1—C3—H3A	117.9	C7—C8—H8A	118.7
C4—C3—H3A	117.9	N2—C9—C10	125.7 (5)
O3—N4—O4	119.7 (6)	N2—C9—H9A	117.2
O3—N4—C7	121.7 (5)	C10—C9—H9A	117.2
O4—N4—C7	118.7 (5)	C9—C10—C6	120.1 (5)
C3—C4—C5	121.0 (5)	C9—C10—H10A	120.0
C3—C4—N3	116.4 (5)	C6—C10—H10A	120.0
			
C3—N1—C2—C1	−2.5 (12)	C4—C5—C6—C7	−72.7 (7)
C5—C1—C2—N1	0.4 (12)	C1—C5—C6—C7	113.3 (7)
C2—N1—C3—C4	3.1 (10)	C10—C6—C7—C8	0.8 (7)
N1—C3—C4—C5	−1.6 (10)	C5—C6—C7—C8	−176.7 (5)
N1—C3—C4—N3	178.8 (6)	C10—C6—C7—N4	180.0 (4)
O1—N3—C4—C3	8.6 (7)	C5—C6—C7—N4	2.5 (8)
O2—N3—C4—C3	−172.1 (6)	O3—N4—C7—C8	162.7 (5)
O1—N3—C4—C5	−171.0 (5)	O4—N4—C7—C8	−17.6 (8)
O2—N3—C4—C5	8.3 (7)	O3—N4—C7—C6	−16.5 (7)
C3—C4—C5—C1	−0.6 (8)	O4—N4—C7—C6	163.2 (5)
N3—C4—C5—C1	179.0 (5)	C9—N2—C8—C7	2.9 (9)
C3—C4—C5—C6	−174.7 (5)	C6—C7—C8—N2	−2.2 (9)
N3—C4—C5—C6	4.9 (8)	N4—C7—C8—N2	178.6 (5)
C2—C1—C5—C4	1.2 (9)	C8—N2—C9—C10	−2.4 (10)
C2—C1—C5—C6	175.9 (6)	N2—C9—C10—C6	1.2 (10)
C4—C5—C6—C10	109.9 (6)	C7—C6—C10—C9	−0.3 (7)
C1—C5—C6—C10	−64.1 (6)	C5—C6—C10—C9	177.4 (5)

### Hydrogen-bond geometry (Å, °)

**Table:**

*D*—H···*A*	*D*—H	H···*A*	*D*···*A*	*D*—H···*A*
C2—H2B···O1^i^	0.93	2.40	3.234 (8)	149
C3—H3A···N2^ii^	0.93	2.62	3.440 (8)	147
C10—H10A···O2^iii^	0.93	2.57	3.392 (6)	148

Symmetry codes: (i) *x*−1/2, −*y*+1/2, *z*+1; (ii) −*x*+3/2, *y*+1/2, *z*−1/2; (iii) *x*, *y*, *z*+1.
